# Authors’ Reply to: Comment on “Facebook as a Novel Tool for Continuous Professional Education on Dementia: Pilot Randomized Controlled Trial”

**DOI:** 10.2196/24084

**Published:** 2020-10-30

**Authors:** Windy SY Chan, Angela YM Leung

**Affiliations:** 1 School of Health Sciences Caritas Institute of Higher Education New Territories Hong Kong; 2 Faculty of Health and Social Sciences The Hong Kong Polytechnic University Kowloon Hong Kong; 3 Centre for Gerontological Nursing School of Nursing, The Hong Kong Polytechnic University Kowloon Hong Kong

**Keywords:** dementia care, social network sites, Facebook

We would like to thank Dr Saishoji and colleagues [1] for the opportunity to respond to the issues raised in their letter and to clarify aspects of our study design in relation to these concerns. We would also like to thank Dr Saishoji and colleagues for their interest in our paper [2] and for taking the time to express their concerns.

Our study, a pilot randomized controlled trial, adopted mixed-research methods (quantitative and qualitative) to evaluate the feasibility and acceptability of utilizing Facebook to deliver a continuous professional education (CPE) program to health care professionals. We believe that it is important to explore different effects of the intervention on participants’ own provision of care. Therefore, we focused on measuring the knowledge relevant for the practical care of people with dementia rather than a high level of scientific knowledge related to dementia [3].

In addition to the primary outcome (knowledge about dementia, via the Dementia Knowledge Assessment Scale), we also evaluated participants’ compliance, participants’ engagement in the intervention, participants’ satisfaction, and participants’ attitudes toward using Facebook for professional education from a quantitative perspective. Although significant differences between the intervention group and the control group were not observed in the primary outcomes, we found that the Facebook intervention did well in offering gains to participants’ knowledge and enhanced their engagement and compliance. For that reason, we tried to elaborate on all the outcomes to bring readers a comprehensive picture of the actual application of Facebook for future CPE programs.

We agree that there may be an increased risk of type 1 error when applying multiple statistical tests, and in some cases, correction, such as the Bonferroni correction, can be used. However, we understand that adopting this kind of correction is not a must; it depends on the rationale of the study [4]. Our study was exploratory, involving a few posthoc comparisons, which were regarded as hypotheses for further investigation. Thus, we believe that no correction would be needed in our pilot study.

We appreciate the comment on how our statistical analysis was reported. Hence, we would like to take this opportunity to provide additional details pertaining to our analysis. In measuring the primary outcomes, an independent sample *t* test was used to compare the changes in the mean knowledge gain scores between the two groups at the postintervention assessments.

We agree with Dr Saishoji and colleagues [1] that online education is intriguing and important during the current COVID-19 pandemic. We would be glad to keep communicating with investigators on research in this area.

## Abbreviations

CPE: continuous professional education

## References

[1] Saishoji Y, Shiroshita A, Tsujimoto Y (2020). Comment on "Facebook as a Novel Tool for Continuous Professional Education on Dementia: Pilot Randomized Controlled Trial". J Med Internet Res.

[2] Chan WS, Leung AY (2020). Facebook as a Novel Tool for Continuous Professional Education on Dementia: Pilot Randomized Controlled Trial. J Med Internet Res.

[3] Tierney L, Mason R, Doherty K, Winbolt M, Long M, Robinson A (2019). Workshops on diagnosis and management of dementia for general practitioners: a pre-post intervention study of dementia knowledge. BMJ Open.

[4] Armstrong Richard A (2014). When to use the Bonferroni correction. Ophthalmic Physiol Opt.

